# The effect of multi-component interventions on the incidence rate, severity, and duration of post open heart surgery delirium among hospitalized patients

**DOI:** 10.1186/s13019-021-01422-0

**Published:** 2021-03-20

**Authors:** Banafsheh Tehranineshat, Nima Hosseinpour, Arash Mani, Mahnaz Rakhshan

**Affiliations:** 1 Community Based Psychiatric Care Research Center, Department of Nursing, School of Nursing and Midwifery, Shiraz University of Medical Sciences, Zand St., Nemazee Sq, Shiraz, 7193613119 Iran; 2 Student Research Committee, School of Nursing and Midwifery, Shiraz University of Medical Sciences, Shiraz, Iran; 3 Psychiatry Department, Research Center for Psychiatry & Behavioral Sciences, Shiraz University of Medical Sciences, Shiraz, Iran , Shiraz, Iran

**Keywords:** Open heart surgery, Delirium, Multi-component interventions, Nursing

## Abstract

**Background:**

Delirium is one of the prevalent complications of post open heart surgery. The present research aimed to assess the effect of multi-component interventions on the incidence rate, severity, and duration of post open heart surgery delirium among hospitalized patients.

**Methods:**

In this quasi-experimental study, 96 patients under open heart surgery were selected using convenience sampling and divided into a control and an intervention group. The interventions included the patients’ preoperative education, nurses’ education, and in-ward environmental interventions. The demographic information and Mini-Mental State Examination (MMSE) questionnaires were completed a day before surgery. The patients in both groups were also surveyed after extubation until the fourth day post operation using Delirium Observation Screening (DOS) scale considering the incidence, severity, and duration of delirium. The data were analyzed using the SPSS statistical software, version 20.

**Results:**

The incidence rate of delirium was 14.6 and 6.2% in the control and intervention groups, respectively (*p* > 0.05). Besides, the mean severity of delirium was 0.53 in the control group and 0.40 in the intervention group (*p* > 0.05). Finally, the mean duration of delirium was 4.5 and 3.25 h in the two groups, respectively (*p* > 0.05).

**Conclusions:**

Since prevention of delirium can play a considerable role in the patients’ recovery after heart surgery, it is necessary to carry out some measures to prevent such complications. Even though the interventions performed in this study did not cause significant changes in this regard, the results suggested that prevention interventions should be performed with stronger and more integrated planning for achieving better outcomes.

## Background

In spite of emphasis on improvement and prevention of risk factors, cardiovascular diseases are still supposed to be the main disability and mortality factor in developing countries [1]. Nonetheless, progress in new therapeutic methods, such as thrombolytic therapy, and interventional methods, such as Percutaneous Coronary Intervention (PCI), has led to improvement in management and treatment of cardiovascular diseases. Yet, surgical interventions are still propounded as the elective treatment for some patients [2], and the number of patients under open heart surgery is increasing daily in industrial countries [3].

Heart surgery may be accompanied by several complications, among which delirium is prevalent with an incidence rate of 30–50% [4]. “Post-surgery delirium” was used by Blachly and Starr for the first time [5]. According to the American Psychological Association, delirium has been defined as a mental transient syndrome characterized by sudden onset and cognitive impairment, decrease in the level of consciousness, attention disorders, increase or decrease in psychomotor activity, and sleep and wake cycle disorder [6]. Delirium and dementia should be differentiated from each other because delirium is characterized by sudden onset, fluctuation, attention disturbance, and disorganized thoughts and speech, while dementia is accompanied by gradual onset, no fluctuations, no inattention, and no speech disturbances. Additionally, delirium is recurrent, while dementia is not [7].

Post-surgery delirium often appears in the first 3 days and mostly on the first day after surgery [8]. The prevalence of post-surgery delirium has been reported to be between 15.0 and 60.0%, but this rate is higher after heart surgery [9]. Delirium is highly prevalent (up to about 80.0%) at Intensive Care Unit (ICU) and may result in an increase in the number of days being under mechanical ventilation, increase in the length of ICU stay and hospital stay, unintentional pulling of tubes and catheters, and mortality [10]. Delirium not only leads to an increase in the length of hospital stay, morbidity, and mortality, but also results in some long-term effects, including functional and cognitive impairment and further hospitalization. Therefore, it imposes a great economic burden on the health system as well as extra expenses up to 60,000 dollars on each patient per year [11]. Hence, some studies mostly using preventive approaches have been carried out to control post-surgery delirium. Accordingly, music therapy and haloperidol-prophylaxis have been introduced as effective methods in prevention of delirium [12].

Up to now, no precise pathophysiology and etiology has been mentioned for delirium, but it has been stated to result from some predisposing and underlying factors [13]. Some of the predisposing factors of delirium include age above 65 years, history of psychotic disorders like schizophrenia, sensory impairment (especially sight), brain injury, history of stroke, sleep deprivation, history of alcohol abuse, prolonged heart and lung bypass, more than one-liter blood transfusion during surgery, hypothermia during surgery and anesthesia, cardiogenic shock, hypoxemia, dehydration, and drugs consumption (psychoactive, analgesic, and hypnotic drugs) [14]. Environmental risk factors are other risk factors, the effects of which on the severity of delirium were shown in a study by Mccusker et al. in [15]. Some of these factors include patient’s isolation, too few or too many sensory stimulations, inappropriate environmental light and sound, and absence of family at patient’s bedside.

Nowadays, researchers have mostly focused on prevention of delirium and the necessity to carry out studies regarding recognition of its risk factors [16]. Some researchers admitted that multi-component interventions could reduce the incidence of delirium among heart surgery patients [17, 18]. However, others have reported that preventive pharmacological interventions do not have significant effects on the incidence of delirium [19]. Based on the previous studies, the main performed interventions were merely related to the post heart surgery period. Also, the incidence rate of delirium was the main outcome evaluated in the previous studies, but the effects of such interventions on the severity and duration of delirium have remained unanswered. Therefore, the present study aimed to survey the effect of multi-component interventions on the incidence rate, severity, and duration of post open heart surgery delirium among hospitalized patients.

## Methods

### Participants and sampling

In this quasi-experimental study, the participants included all the patients who had undergone open heart surgery at Kosar Hospital (Fars Heart Foundation), Shiraz, Iran in February to November 2019. The participants were selected through convenience sampling and in case they fulfilled the inclusion criteria of the study and signed written informed consents, they were recruited in the study. The sample size was calculated to be 42 subjects (in each group), based on the results of the study of Karimi et al. [20] and the following measures in the formula below: α = 5%, statistical power = 90%, confidence interval = 95%, μ1 = 28, μ2 = 26.62, σ1 = 1.21, and σ2 = 2.46.

$$ n=\frac{{\left({Z}_{1-\frac{\propto }{2}}+{Z}_{1-\ss}\right)}^2\kern0.5em \left({\sigma}_{1\kern0.5em }^2+{\sigma}_{2\kern0.75em }^2\right)}{{\left({\mu}_1-{\mu}_2\right)}^2} $$

Considering a 15% chance of loss to follow-up, 48 patients were assigned to each group, bringing the total to 96. To ensure that the groups were homogeneous, the same inclusion and exclusion criteria were applied to both groups. Initially, starting from a certain date, subjects were selected through convenience sampling for the control group. The subjects in the control group only completed the demographics questionnaire and the MMSE and DOS scales—neither the patients not the personnel received any education and no environmental interventions were introduced. After the last subject in the control group had been discharged, the researchers sampled subjects for the intervention group and applied the multi-component interventions, consisting of education for the nursing staff, education for the patients, and environmental interventions.

The demographic information questionnaire was completed by all the participants; then, they were divided into control and intervention groups. Since two similar research environments were not available simultaneously and the interventions caused environmental changes as well as changes in the process of patients’ caretaking, first 48 individuals from the control group and, then, 48 subjects from the intervention group underwent investigation. The inclusion criteria of the study were age under 65 years, lack of a history of debilitating mental illness and neurological diseases, a minimum Mini-Mental State Examination (MMSE) score of 25.0, no addiction to drugs and alcohol, and the ability to communicate. On the other hand, the exclusion criteria of the study were mechanical ventilation for more than 12 h, heart and lung bypass for more than 90 min, and repeated heart surgery for any reason, such as bleeding and tamponade.

### Measurement instruments

The applied instruments in this study were as follows:

1. Demographic information form, which was prepared based on a review of the literature and experts’ opinions. This form included sex, age, place of residence, education level, occupation, history of underlying diseases, and type of surgery.
2. MMSE, which contained 11 questions with a total score between 0 and 30. This scale evaluated cognitive defects and changes over time and could be completed within only 5–10 min [21]. In order to investigate the psychometric properties of this instrument in Iranian society, Cronbachʼs alpha coefficient method (0.87) and split-half technique (0.71) were used. In addition, the validity of MMSE was assessed by concurrent criterion method, revealing a significant inverse relationship between the score of this test and that related to the Global Deterioration Scale (GDS) (*p* = 0.0001). The sensitivity and specificity of the questionnaire were also determined as 0.90 and 0.84, respectively [22].
3. Delirium Observation Screening (DOS) scale, which consisted of 13 items. The maximum total score of this scale was 13, with scores equal to or more than 3, representing the incidence of delirium. DOS scale was used in this study because it is made for the nurses, can be completed in less than 5 min, and provides the possibility to determine the severity of delirium. The ability to assess the severity of delirium is yet another strong point of this instrument [23]. In this study, DOS scale was scored three times a day by nurses via observation, and the final score was computed as the mean of the three scores. The validity of DOS scale was surveyed by Delirium Rating Scale-Revised-98 (DRS-R-98); it was estimated to be 0.67. Additionally, its reliability was assessed by Inter Class Correlation (ICC); it was reported to be 0.99 [23, 24]. In order to assess the psychometric properties of DOS scale in Iranian society, we firstly translated this instrument into Persian by an expert translator. Thereafter, in order to determine its content validity, we consulted 11 experts to express their opinions regarding the relevancy, clarity, and simplicity of the items using Waltz and Bausell validity index. Face validity of the instrument was also assessed by 6 nurses who worked at ICU of heart surgery ward and were nursing experts. Moreover, the reliability of DOS scale was evaluated using inter-rater reliability method. In doing so, the instrument was completed for 13 heart surgery patients by the researcher and an educated nursing expert. Then, the correlation between the scores produced by the two observers was calculated, and the results indicated an acceptable reliability (*r* = 0.83).

### Intervention

The multi-component intervention consisted of three parts, namely training the nursing personnel, training the patients, and environmental interventions. Training the personnel of heart surgery ICU was carried by the researcher in the form of a two-hour session. Considering the personnel’s rotational shifts and the necessity of their presence, this class was repeated for three times. In this way, training the personnel regarding delirium and its importance, methods of prevention and treatment of delirium, correct way of communicating with such patients, and orienting patients about time, place, and person was carried out at least twice in each shift. The educational content was presented through lecture and discussion. Moreover, the presented materials were installed as a poster at the nursing station so as to increase the personnel’s exposure to the subjects.

Pre-surgery training of the patients was carried out by the researcher at least 24 h before the open heart surgery. This was done via individual face-to-face conversations along with some pictures from ICU for a period of at least 15 min. During this conversation, in addition to education, the patients’ questions and ambiguities were answered. The preoperative educational content consisted of type and process of the disease, surgery method, time of transfer to the ICU after surgery, environment and equipment of ICU, possible limitations such as inability to talk or drink water due to the existence of Endo Tracheal Tube (ETT) and complete rest on the bed, approximate length of ICU stay, limitations related to visiting one’s family at the ICU, reassuring the patients regarding pain monitoring and control by the personnel, independence from analgesic drugs, and all the therapeutic and nursing measures that were going to be carried out during the days after the surgery.

In addition to training the personnel and patients, environmental interventions were also conducted in this study. These interventions consisted of light adjustment, using calendar and clock, and providing the patients with the possibility to visit their families at the ICU for 10 min once a day and making phone calls to their family members at least once at each shift. Using communication aids, such as glasses, hearing aids, and dentures, for the patients who were using such equipment before the surgery, rapidly sending the patients out of bed, and preventing dehydration by assessing the tongue mucus and skin turgor, as one of the risk factors of postoperative delirium, were among the other interventions carried out for the patients.

### Design

Since physical environment of the ICU can influence the study results, it was necessary to carry out the investigation on both control and intervention groups at one ward in order to make a similar research environment. Therefore, both control and intervention groups were studied at a single ward, but at two different times. Moreover, since the interventions could cause environmental changes as well as changes in the process of patients’ caretaking, first the control group and then the intervention group underwent investigation.

The patients in the control group received the routine care at the post open heart surgery wards of Shiraz Kosar Hospital and were assessed concerning the incidence, severity, and duration of delirium once at the end of each shift until the end of the fourth day after surgery. It should be mentioned that the incidence and severity of delirium was assessed using DOS scale by some fixed in-charges at the ward because at least one of them was present at each shift. Additionally, since they did not play any direct roles in the patients’ caretaking and doing postoperative interventions, they had no knowledge about the control and intervention groups. Before completing the DOS scale, the necessary information about using this instrument was given to the nurses in a one-hour session. Thereafter, their ability in using the instrument was evaluated by putting them at a similar position, i.e. patient’s bedside. After making sure about their knowledge and ability in using the DOS scale, they assessed the incidence and severity of delirium. In the control group, the incidence, severity, and duration of delirium were evaluated by the researcher and trained nurses using the DOS scale after the patients’ consciousness and extubation thrice a day at the end of each shift (8 A.M., 2 P.M., and 8 P.M.) until the end of the fourth day after the surgery.

After completion of sampling in the control group, the other group of participants who met the inclusion criteria and signed written informed consents were entered into the intervention group. Then, the systematic interventions, including training the nursing personnel, training the patients, and environmental interventions, were performed. Also, in this group, the incidence, severity, and duration of delirium were evaluated as in the control group.

### Statistical analysis

After all, the collected data were analyzed in the SPSS statistical software, version 20 using independent t-test, chi-square test, one-way ANOVA, and Pearson’s correlation coefficient.

## Results

This study was carried out on 96 patients (48 in the control group and 48 in the intervention group) who had undergone open heart surgery. The results of chi-square test indicated that the patients in the control and intervention groups were similar with respect to sex, place of residence, education level, occupation, history of suffering from underlying diseases, and type of surgery (Table 1). Additionally, the results of independent t-test showed no significant difference between the two groups regarding age, MMSE score, length of mechanical ventilation, and length of heart and lung bypass (Table 2).

**Table 1:** Frequency distribution of qualitative variables in the control and intervention groups

Group		Control		Intervention		*P*-value
Variable		Frequency	Frequency percentage	Frequency	Frequency percentage	
Sex	Female	17.0	35.4	11.0	22.9	0.26
	Male	31.0	64.6	37.0	77.1	
Place of residence	The capital (Shiraz)	12.0	25.0	9.0	18.8	0.34
	Cities	24.0	50.0	31.0	64.6	
	Villages	12.0	25.0	8.0	16.7	
Level of education	Illiterate	9.0	18.8	8.0	16.7	0.96
	Primary school	11.0	22.9	12.0	25.0	
	High school	5.0	10.4	5.0	10.4	
	Diploma	17.0	35.4	19.0	39.6	
	Academic	6.0	12.5	4.0	8.3	
Occupation	Self-employed	15.0	31.3	16.0	33.3	0.64
	Employed	6.0	12.5	3.0	6.3	
	Unemployed	1.0	2.1	2.0	4.2	
	Retired	14.0	29.2	18.0	37.5	
	Homemaker	11.0	22.9	8.0	16.7	
	Student	1.0	2.1	0.0	0.0	
History of underlying diseases	Diabetes	2.0	4.2	3.0	6.3	0.57
	Hyperlipidemia	5.0	10.4	4.0	8.3	
	Hypertension	6.0	12.5	10.0	20.8	
	More than one Disease	27.0	56.3	22.0	45.8	
	None	8.0	16.7	9.0	18.8	
Type of surgery	Coronary artery bypass graft	38.0	79.2	40.0	83.3	1.0
	Repair/replace of the valves or other types of surgeries	10.0	2.8	8.0	16.7

**Table 2:** Comparison of the means of quantitative variables in the control and intervention groups

Group		Mean	Standard deviation	P-value
Variable				
Age	Control	53.5	9.97	0.37
	Intervention	55.19	8.52	
Mini-mental state examination	Control	29.6	1.04	0.76
	Intervention	26.67	0.99	
Length of mechanical ventilation	Control	10.1	1.75	0.5
	Intervention	9.88	1.59	
Length of heart and lung bypass	Control	69.79	15.15	0.41
	Intervention	67.40	13.48	

The results of chi-square test also revealed no significant difference between the control and intervention groups concerning the incidence of delirium (Table 3). Besides, the mean severity of delirium was 0.53 + 1.43 in the control group and 0.40 + 1.20 in the intervention group; the results of independent t-test showed that the difference was not statistically significant (*p* > 0.05). Finally, the mean duration of delirium was 4.5 + 13.86 h in the control group and 3.25 + 13.27 h in the intervention group, and the results of independent t-test demonstrated that the difference was not statistically significant (*p* > 0.05).

**Table 3:** Frequency distribution of incidence of delirium in the control and intervention groups

Group		Control group		Intervention group		*P*-value
Variable		Frequency	Frequency percentage	Frequency	Frequency percentage	0.31
Incidence of delirium	Yes	7.0	14.6	3.0	6.2	
	No	41.0	85.4	45.0	93.8	

## Discussion

The present study aimed to investigate the effect of multi-component interventions on the incidence rate, severity, and duration of post open heart surgery delirium among 96 hospitalized patients. The study results showed that multi-component interventions, including training the patients and the nursing personnel and environmental interventions, had no effects on the incidence rate, severity, and duration of delirium.

According to the results, although the incidence of delirium in the intervention group (3 cases) was lower than that in the control group (7 cases), the difference was not statistically significant. Similarly, Bakker et al. [25] conducted a study to assess the effect of The CareWell in Hospital program on functional status of hospitalized elderly patients and found that the preventive interventions had no effects on the incidence rate of delirium. In contrast, Mansoori et al. [26] performed a research on hospitalized patients at Coronary Care Unit (CCU), indicating that modification of sensory stimulations led to a significant reduction in the incidence of delirium in the intervention group compared to the control group. The difference between the results of the present study and the one conducted by Mansoori et al. might be due to the fact that Mansoori et al’s study was done in CCU wards, while ours was performed in ICU after heart surgery. In addition, in the study by Mansoori et al., the patients in the control and intervention groups were at two different wards and, consequently, different personnel and physical environments could influence the results. In the present study, on the other hand, both groups were at a single ward, but underwent investigation at two different times.

Zolfaghari et al. [17] also investigated the effectiveness of multifactorial interventions in prevention of the incidence of delirium and length of hospital stay among heart surgery patients. The results revealed that these interventions led to a decrease in the incidence of delirium, but did not have any effects on the length of hospital stay [17]. The difference between the results of the research by Zolfaghari et al. and the current one might be attributed to the fact that a different instrument (CAM-ICU) was used in that study to assess delirium. Besides, unlike the present study, the patients exposed to the risk of delirium (including those with prolonged mechanical ventilation and heart and lung bypass) were not excluded from that study.

The findings of our study showed that the mean severity of delirium was 0.53 in the control group and 0.40 in the intervention group, but the difference was not statistically significant. These results were similar to those of the study conducted by Watne et al. [27], which aimed to investigate the effect of multi-component interventions on prevention of delirium in hospitalized older patients. The results of that study indicated no significant difference between the control and intervention groups regarding the severity of delirium.

The findings of the current study revealed that duration of delirium was 4.5 h in the control group, which reduced 3.25 h in the intervention group. However, the difference between the two groups was not statistically significant. Bjorkelund et al. [28] and Deschod et al. [29] carried out a study to reduce delirium after hip fracture and showed no significant difference between the intervention and control groups regarding the duration of delirium after the interventions.

Overall, difference among the results of various studies in this field could be related to differences in the applied instruments for assessment of delirium, different physical environments, differences in the nature of the participants’ diseases, not considering the effective factors in the incidence of delirium as the inclusion and exclusion criteria, and differences in therapeutic methods used for management of delirium. In the present study, multi-component interventions did not result in a significant reduction in the incidence, severity, and duration of delirium after heart surgery. However, if the number of the study patients and cases with delirium in the control group was more, different results might have been obtained.

One of the limitations of this study was the rotational shift of resident anesthesiologists at the ICU, because various physicians’ therapeutic methods for management and treatment of delirium can influence the results. Moreover, delirium in this study was assessed three times during 24 h. Therefore, the symptoms of delirium or suffering from fluctuation in the severity of delirium at other times were not considered in this study, which could have affected the results. Hence, further studies with larger sample sizes and elimination of the aforementioned limitations are recommended to be conducted on the issue.

## Conclusions

Given that reduction in the incidence, severity, and duration of delirium can play a considerable role in the patients’ recovery after heart surgery, it is necessary to take measures to prevent delirium. However, the interventions carried out in the present study did not lead to significant changes in this regard. Therefore, stronger and more integrated preventive interventions should be performed to improve such patients’ recovery process.

## Data Availability

The data will not be shared since the participants are guaranteed full anonymity.

## Abbreviations

MMSE: Mini-Mental State Examination
DOS: Delirium Observation Screening
PCI: Percutaneous Coronary Intervention
ICU: Intensive Care Unit
CCU: Coronary Care Unit
GDS: Global Deterioration Scale
DRS-R-98: Delirium Rating Scale-Revised-98
ICC: Inter Class Correlation
ETT: Endo Tracheal Tube
